# Italian inter-society expert panel position on radiological exposure in Neonatal Intensive Care Units

**DOI:** 10.1186/s13052-020-00905-5

**Published:** 2020-10-27

**Authors:** Antonella del Vecchio, Sergio Salerno, Massimo Barbagallo, Gaetano Chirico, Mauro Campoleoni, Vittorio Cannatà, Elisabetta Genovese, Claudio Granata, Andrea Magistrelli, Paolo Tomà

**Affiliations:** 1 Associazione Italiana di Fisica Medica (AIFM), Milan, Italy; 2 Servizio di Fisica Sanitaria, IRCCS Ospedale San Raffaele, Via Olgettina, 60, 20132 Milan, Italy; 3 Società Italiana di Radiologia Medica e Interventistica (SIRM), Milan, Italy; 4 Società Italiana di Pediatria (SIP), Milan, Italy; 5 Società Italiana di Neonatologia (SIN), Milan, Italy

**Keywords:** Radiology, Radioprotection, Intensive care unit, Paediatric exposure

## Abstract

**Background:**

In the recent years, clinical progress and better medical assistance for pregnant women, together with the introduction of new complex technologies, has improved the survival of preterm infants. However, this result requires frequent radiological investigations mostly represented by thoracic and abdominal radiographs in incubators. This document was elaborated by an expert panel Italian inter-society working group (Radiologists, Paediatricians, Medical Physicists) with the aim to assist healthcare practitioners in taking choices involving radiation exposures of new-born infants and to provide practical recommendations about justification and optimization in Neonatal Intensive Care Units. The adherence to these practice recommendations could ensure a high quality and patient safety. More complex and less common radiological practice, such as CT scan or fluoroscopy have been excluded.

**Methods:**

The consensus was reached starting from current good practice evidence shared by four scientific societies panel: AIFM (Italian Association of Physics in Medicine), SIN (Italian Neonatology Society), SIP (Italian Paediatric Society), SIRM (Italian Medical Radiology Society) in order to guarantee good standard practices for every professional involved in Neonatal Intensive Care Units (NICU).

The report is divided into clinical and physical-dosimetric sections: clinical Indications, good practice in radiological exposures, devices, exposure parameters and modalities, patient positioning and immobilization, Reference Diagnostic Levels, operators and patient’s radiation protection.

Another important topic was the evaluation of the different incubators in order to understand if the consequences of the technological evolution have had an impact on the increase of the dose to the small patients, and how to choose the best device in terms of radiation protection.

At the end the working group faced the problem of setting up the correct communication between clinicians and parents following the most recent indications of the international paediatric societies.

**Results:**

Taking into account the experience and expertise of 10 Italian Centres, the guideline sets out the criteria to ensure a high standard of neonatal care in NICU about procedures, facilities, recommended equipment, quality assurance, radiation protection measures for children and staff members and communication on radiation risk.

**Conclusions:**

This document will allow a standardization of the approach to the exposures in NICU, although oriented to a flexible methodology.

## Introduction

In the last decades, the clinical progress, the better medical assistance for pregnant women even in very difficult gestations, together with the introduction of new complex technologies, allowed the improvement of preterm, and/or with serious disease infants, survival. Nevertheless, the survival increase in such high-risk infants frequently requires radiological surveys, mainly chest- and abdomen-x ray in incubator.

In order to get better safety levels, it is a common opinion to share different specialist experiences in order to define best practice recommendation and guidelines [1–3].

The aim of this document is to assist healthcare practitioners in taking choices involving radiation exposures of new-born infants and to give practical recommendation about justification and optimization in Neonatal Intensive Care Units. These expert panel was constituted by expert from four Italian main representative scientific societies: AIFM (Italian Association of Physics in Medicine), SIN (Italian Neonatology Society), SIP (Italian Paediatric Society), SIRM (Italian Medical Radiology Society) in order to guarantee good standard practices for every professional involved in Neonatal Intensive Care Units (NICU). The adherence to these practical recommendations could ensure a high quality and patient safety. More complex radiological practice, such as CT scan or fluoroscopy have been excluded.

## The expert panel

The panel was constituted by experts from the main society on the field including paediatric radiologist, neonatologist, paediatricians, medical physics and radiologist with expertise in radiation protection. There are new evidence and regulation in radiation protection and a relative lack in radiation protection culture among clinicians. There is also a relative lack of consensus in the published literature as to the best approach to use to communicating risks to patients and relatives. The methodology used was: 1) recognized common clinical request for performing a radiological procedure; 2) good practice for the execution of the radiographic exams in NICU with a multidisciplinarity to facilitate diversity of views and expertise from paediatric radiologist, neonatologist, paediatricians, medical physics and radiation protection perspective; and 3) both a national and international recommendation (ICRP), European directive (EURATOM) context to facilitate a global representation and exchange of state-of-the-art knowledge, with the aim of implementing execution of the radiographic exams in NICU [4–6].

## Clinical indications

The clinical indications for carrying out investigations that require the use of ionizing radiation in new-borns admitted to neonatal intensive care unit (NICU), and assisted in an incubator, are varied and mainly divided into prescriptions for the study of the thorax and abdomen.

### The most frequent thoracic indications are [7–9]

- Neonatal respiratory distress syndrome (NRDS), which generally affects the preterm infant, and is secondary to a surfactant deficiency and to the immaturity of the respiratory system and alveolar capillary unit.
- Congenital abnormalities of surfactant synthesis.
- Transient tachypnoea of the new-born (or “wet lung disease”) (TTN), a condition resulting from the abnormal retention of foetal lung fluid, generally found in births by elective caesarean delivery, or in “late preterm” new-borns, with 34–36 weeks gestational age.
- Meconium aspiration syndrome (MAS), which causes respiratory failure through various mechanisms, such as airway obstruction, infection, chemical irritation and surfactant inactivation.
- Bacterial, viral or fungal pneumonia can be considered, like the previous conditions, a real emergency, especially in preterm and low birth weight infants.
- Malformations (es, congenital diaphragmatic hernia (CDH), oesophageal atresia and tracheoesophageal fistula (TEF), congenital pulmonary airway malformation (CPAM), congenital heart diseases), before and after surgery.
- Bronchopulmonary dysplasia (BPD).
- Complications of mechanical ventilation or “air leak syndrome” (es, pneumothorax, pneumomediastinum, pulmonary interstitial emphysema (PIE)).
- Pleural or pericardial effusions in hydropic new-borns or following parenteral solutions extravasation.
- Suspected fractures or malformations of the thoracic skeleton.
- Prenatal ultrasound finding of suspected or confirmed anomaly.

In these situations, only one radiogram in the anterior-posterior projection (AP) is generally sufficient; additional radiographic projections may be required whenever pneumothorax and/or pneumomediastinum are suspected.

However, in the context of the aforementioned clinical indications the development of pulmonary ultrasound should be considered [10–12]. There is no need to perform chest radiographs, without clear clinical indication, in case of or for [7–9, 13]:

- daily examination in new-borns with mechanical ventilation;
- pre- and post-intubation;
- each re-intubation to confirm the endotracheal tube position;
- any worsening of the value of SpO2 or any situation in which increasing the amount of O2 to be supplied to the new-born is needed;
- mild respiratory distress.

### Abdominal indications

May occur in new-borns with prenatal ultrasound findings of suspected or confirmed anomaly, or with alterations of intestinal transit, which have gastric residual, vomiting, abdominal distension [14, 15].

In neonates with proximal occlusion and non bilious vomiting, a gastric atresia, congenital antral membrane, pyloric atresia or duodenal atresia should be suspected. If the vomit is bilious duodenal atresia or stenosis, malrotation or preduodenal portal vein can be the possible causes. In these cases radiograph in AP projection and ultrasound study are able to provide the correct diagnosis. In the suspicion of antral membrane or intestinal malrotation completing the investigation with oral administration of a low-osmolality contrast medium is opportune.

In the case of new-borns with low occlusion and abdominal distension, in which a jejunoileal or colic pathology can be observed, the direct radiogram of the abdomen in anterior-posterior projection and a radiological study with endorectal low-osmolarity contrast medium shows the localization of upstream gas distension and a non-use microcolon pattern, respectively. In the anal atresia or imperforation, it is useful to request the invertogram with radio-opaque marker.

A separate condition is necrotizing enterocolitis (NEC), which can develop in preterm and low birth weight new-borns, and is characterized by pneumatosis intestinalis, necrosis and perforation. The radiogram in AP projection shows the signs of intestinal distension, intestinal and portal pneumatosis and, in the advanced stage, abdominal free air. If intestinal perforation is suspected, the lateral projection may be useful, in the supine position or in the left lateral decubitus.

Radiograms of the abdomen do not need to be performed without clear clinical indication [16] in:

- gastroenteritis;
- hematemesis;
- hypertrophic pyloric stenosis (HPS).

### Other indications

Are the verification of the correct positioning of endotracheal tubes, pleural or peritoneal drainages, venous or arterial lines (umbilical and central). In such cases, the AP projection of the thoracic or abdominal area to be examined is generally sufficient. However, the development of ultrasound in these areas must be taken into consideration [17].

## Good practice for the execution of the radiographic exams in NICU

The implementation of the patient radioprotection principles, as reported in the national and international low [18, 19], foresees that all the individual medical exposures must be preliminarily justified, keeping in mind the specific objectives of the exposure, the patient characteristics and the diagnostic path that the patient is completing.

Following the choice of a considered appropriate exam, it is necessary to respect the optimization principle, that requires the implementation of all the shrewdness to maintain the doses as low as possible in accordance with a correct exam result [18, 19].

It means to use appropriate radiological devices subjected to a periodic Quality assurance program in line with the current law on patient protection [19]. Moreover, some critical issues that can increase the patient dose [2] should be prevented:

- wrong patient positioning;
- optical field inaccurate or not present on the equipment;
- devices and execution protocols not well known because of the workers turnover;
- presence of devices or materials that could pollute the images;
- mechanical fragility and resulting in position instability;
- detectors impairment due to an accidental fall;
- artifacts due to poor detector cleaning.

### Appropriateness and quality of the equipment

In a NICU, it is necessary to have a portable radiographic equipment that provides the best patient safety and adequate functionalities to produce radiographs with excellent diagnostic image quality in terms of acquisition and post-processing parameters, together with very short exposure times, suitable for new-borns. The image acquisition modality must be based on indirect digital detectors (CR, “computed-radiography”) or direct (DR, “digital-radiography”).

The latter method is preferable due to the increased sensitivity, dose reduction, speed and ease of use.

It is also strongly recommended that the radiological equipment is equipped with a system that tracks and displays the delivered dose (P_KA_ or DAP, dose-area product) at the time of exposure.

It is advisable that the department has always access to the same equipment on which the radiographers have carried out a thorough training.

It is also recommended that not only the current diagnostic reference levels (LDR) are respected, but also that a comparison is carried out considering the most recent recommendations found in international guidelines, which are constantly updated, and the specialist literature [20].

### Patient, examination and diagnostic images identification

Generally, medical structures have internal procedures meant to correctly identify patients, based on national and/or international guidelines. Below in this document, some good practice recommendations are provided [21–23].

Operators shall verify the new-born’s identity and the correspondence between the patient and the requested examination before every radiologic practice. Nowadays, these operations are simplified because the NICU (Neonatal Intensive Care Unit) issues identification bracelets that facilitate the patient’s identification and the correspondence between personal details related to the examination request and the patient itself. Such data and position indicators (left and right) shall be clearly visible in the diagnostic image [21].

### Patient positioning and immobilization

The correct patient positioning is essential for a successful radiographic examination and is one of the crucial factors to avoid repeated and undue exposures for the patient. Immobilization can be obtained through the use of some instruments, such as synthetic materials “nests” or paediatric sandbags. The radiographer provides nurses with all the indications about the aforementioned instruments positioning to avoid new-born incorrect position as well as interposition of medical devices within the field of investigation.

In order to reduce risks related to the onset of infections or other problem for the new-born and to assure the diagnostic examination reproducibility, a good practice recommendation is to use, for the radiologic examination, a cassette (where available).

Should the cassette be unavailable or in case of specific examination requirements, it is possible to perform the x-ray examination through the detector physically contacting the new-born [21–23]. In such cases the detector must be wrapped in a polyethylene bag or similar, preferably sterile, in order to ensure the maximum safety and to limit infection risks. Exceptionally, new-borns may be immobilized by the operator, that shall be equipped with protection device from diffuse radiation and shall remain outside the primary beam. This task is strictly forbidden for women whose pregnancy Is ascertained.

### Size of the field of view and collimation

Correct beam limitation to the field of interest is paramount. X-ray field size must never be larger than necessary, at the same time including the anatomy of interest in order not to compromise the diagnosis or make a second exposure necessary (doubling the dose).

It is mandatory to limit the number of Chest & Abdominal Radiographies (single radiogram) [2, 3, 7–9].

The “Chest & Abdominal “with a unique exposure can be useful, in selected cases, to verify the position of catheters/lines/tubes. We emphasize that focusing the beam on the abdomen involves a difficult reading of the thorax, which appears with a pseudo lordotic image (oblique beam).

Collimation should include:

Chest Radiography in Neonates.

- Superiorly: at the level of the chin, nothing above this level should be irradiated.
- Inferiorly: just below the level of the nipples.
- Laterally: to include the lateral chest walls.

Abdominal Radiography in Neonates

- Superiorly: to include the top of the diaphragm.
- Inferiorly: to symphysis pubis.
- Laterally: to include the lateral abdominal walls.

Chest & Abdominal Radiography in Neonates.

- Superiorly: at the level of the chin, nothing above this level should be irradiated.
- Inferiorly: to symphysis pubis.
- Laterally: to include the lateral chest and abdominal walls.

In children, 40% of the red marrow is inside the long bones and in the skull. It is therefore necessary to respect the above-mentioned collimations, not including the limbs or the head-neck in the radiograms. In cases where it is mandatory to reveal the folding back of the catheters, the insertion point must be included in the radiogram.

### Radiation shielding

The exposure of the gonads of the new-born, especially preterm, raises concerns about the potential long-term harmful effects. Generally, the positioning of shields for the male gonads is complicated because, especially in premature infants, the gonads are found near to the pubic symphysis and is very difficult to exclude them from the field of view of radiograms. In the case of the female gonads, however, it is very difficult to shield the ovaries without covering the entire pelvis. The entrance dose accepted for neonatal abdominal radiography is generally very low and the probability of occurrence of long-term effects due to this exposure is negligible [24]. If you use proper collimation, a correct filtration addition and proper positioning of the patient, the dose to the gonads of the new-born, in the absence of lead shielding, is in the order of about a few tens μGy for males; for females it is further reduced because the ovaries are naturally shielded by 3 to 4 cm of soft tissue. The expected benefit from the use of shielding is not offset by the potential risk of obscuring the patient’s anatomical structures and repeating the examination.

### Parameters and exposure mode

The exposure depends on:

- The method of execution with a detector in contact with the new-born or inserted in the cradle-box holder determines important differences in the delivered dose to the disadvantage of the second;
- the detection system employed: DR systems today are those that allow the best ratio between image quality and dose.

Furthermore, the presence of additional filters should be considered. Optimization is possible only by studying the available system, and overall, the applied voltage can range from 50 to 65 kVp and 0.5 to 3 mAs with a focus-receptor distance (DFR) set at 100 cm. If possible, the patient should be exposed during the inspiration phase when the examined area is in the chest, and expiration in case the examined area is that of the abdomen alone [9].

### Diagnostic reference levels

The latest diagnostic reference levels (LDR) are those proposed by the European project PiDRL [25] which is currently being published. This document, however, only shows, for the new-born, the indication of P_KA_ of 15 mGy*cm^2^ for a AP chest radiograph, while for other views it is suggested to refer to other literature data. The most recent ones include the values in Table 1.

**Table 1:** Most recent DrL

*Author*	*year*	*View*	*DRL or proposal*
Veit [26]– DE	2010	AP-PA Chest premature (approx. 1 kg) AP-PA chest newborn (approx. 3 kg)	**P**_**KA**_**:** 3 mGy*cm^2^ **P**_**KA**_**:** 5 mGy*cm^2^
PiDRL [25] – EU	2016	AP-PA chest (< 10 kg)	**P**_**KA**_**:** 15 mGy* cm^2^
Bahreyni Toossi [27] – IR	2012	Chest abdomen	**K**_**a,e**_: 88 μGy **K**_**a,e**_: 98 μGy
Billinger [28] – AT	2010	Chest	**K**_**a,e**_: 50 μGy **P**_**KA**_**:** 17 mGy*cm^2^
		Abdomen	**K**_**a,e**_: 200 μGy **P**_**KA**_**:** 60 mGy*cm^2^

### Examples of good radiological practice in neonatal radiology

The clinical suspicion must always be reported in the prescription of the examination in order to document the appropriateness of the latter for that specific case [9].

#### Chest X-ray with anteroposterior projection

Diagnostic quality requirements:

- Symmetrical image of the chest without rotations
- Chest image including the cervical portion of the trachea up to T12/L1
- Visualization of the vascular pattern of the lung
- Visualization of the trachea and proximal bronchi
- Clear visualization of the diaphragm and costophrenic angles
- Visualization of the vertebral column and paravertebral structures
- Visualization of the retrocardiac lung and mediastinum

#### Abdomen X-ray with vertical/horizontal beam

Diagnostic quality requirements [9]:

- Visualization of abdomen, diaphragm including ischiatic tuberosities, and abdominal lateral walls
- Visualization of intestinal meteorism and content
- Clear visualization of bones.

When intestinal perforation or bowel obstruction is suspected, additional X-ray with lateral projection.

## Type of incubators and impact on exposure

The risk of infections is to be considered among the main fatalities in the process of treating preterm babies.

This is one of the reasons that have led the manufacturers of specific NICU incubators to produce cots with a box-holder placed under the bed. In this way the cassette never comes in contact with the child and the risk of infections is reduced.

As a consequence, this higher focus-detector distance forces to increase exposure parameters and leads to a higher patient dose value that must be considered when purchasing these devices.

Another recent modification of commercially available NICU incubators consists of interposing a scale between the child and the cassette-holder, and this allows the medical staff to carry out a constant monitoring of the weight of the new-born, keeping the patient isolated in a protected environment [9, 29].

All these changes have certainly been designed to benefit the new-born patient, however, from the standpoint of radioprotection, we must remember that whatever material is interposed between the patient and the image receptor, it leads to a dose increase in patients who might weigh even less than one kilogram and are usually subjected to a remarkable number of tests, due both to the seriousness of their health condition and to the length of the period of hospitalization [29].

To quantify to what extent these changes have influenced the dose increase, a measurement campaign was carried out in 10 Italian hospitals and the data have been elaborated by AIFM. The following is a summary of the results obtained.

The method used to calculate the attenuation was as follows:

- Measurements taken at different energy values (50, 55, 60, 65, 70 kVp) were considered;
- The attenuation was obtained comparing measurements of the beam attenuated by all the material in the beam, canopy with support and mattress, and by the canopy only.

In general, it can be said that depending on the model, with a typical X-ray beam inherent filtration (1.5–2 mm Al) the attenuation in the range 50–70 kVp varies between 20 and 25% on average, while in case of use of additional filters, typically 1 mm of aluminium (Al) and 0.1 mm of copper (Cu), the attenuation decreases to 16 and 20% on average. The use of additional filters is therefore always recommended.

In the incubators in which the inserted weighing scale results in a further attenuating factor, this is of the order of 40–50% without additional filtration, while it is of the order of 15% with added filtration, it would therefore be advisable to avoid incubators with metal supports as they attenuate the X-ray beam more than all the others, even with added filtration.

## Patient and NICU operators radioprotection

This section provides quantitative information about exposure to ionizing radiation levels in a NICU: i) other patients, close to the one involved in the radiologic examination, ii) accompanying persons and iii) the ward staff. Moreover, the protection devices efficiency (e.g. mobile bulkheads and anti-X sheet) will be evaluated in the following [2, 18, 19].

During the examination, hospitalized patients cannot be moved out of the ward. For this reason, during the irradiation, the other patients in the room are exposed to the diffuse beam coming from the under-examination patient close to them.

In order to investigate the diffuse radiation effect, there have been some tests performed and are described as follows: the measurement of radiation diffused from the cradle involved in the examination has been achieved at different angles and from 1 mt of distance both for AP and LL projections with a mobile radiographic device equipped with CR image detector: 60 kVp, 0.5 mAs (50 mA and 0.01 s) for AP projection; 70 kVp, 0.5 mAs (50 mA and 0.01 s) for LL projection.

The obtained values for a single radiogram oscillate between 0.01 ÷ 0.05 μSv/radiogram for the AP projection and between 0.03 ÷ 0.50 μSv/radiogram for the LL projection. Supposing that 90% of the radiograms are performed in AP projection and the remaining 10% in LL projection, the values for single radiogram oscillate between 0.012 ÷ 0.095 μSv/radiogram.

Assuming that a patient has n. 4 adjacent cradles and that each of the close patients performs n. 1 radiogram/day (90% in AP and 10% in LL) for 365 consecutive days, the annual dose value due to the diffuse radiation at 1 my distance is in the order of 150 μSv/year. The dose absorbed for a patient at 1 mt distance during a solar year is therefore well below 500 μSv.

It is then not deemed necessary to use mobile screens (e.g. bulkheads) if the incubators are at least at 1 mt of distance from each other. The efficacy of anti-X sheets for reducing the diffuse radiation has been investigated using the same geometry described above. A Pb equivalent anti-X sheet (0.25 mm thick) has been used to cover the cradle adjacent to the one involved in the examination. Although the use of protection devices such as anti-X screens allows a 100 times lower dose reduction compared to an already low value, its use is not recommended because the expected benefit is not balanced by the risk due to loss of visual contact with the patient and interference with vital parameters monitoring equipment (taking into account the optimization principle) [9–19].

During the irradiation of a patient undergoing examination, the accompanying persons and any staff member present may also be exposed to the diffuse radiation. The results of environmental dose assessments in a typical NICU ward, obtained by passive environmental dosimetry, report dose values lower than the natural radiation background. For distances greater than 1 mt from the axis of the primary beam, the dose absorbed by a hypothetical individual exposed during an entire calendar year is less than the value of 1 mSv/year, equal to the exposure limit to ionizing radiation for the population currently provided for in the Italian law in force. Since the absorbed dose decreases at the inverse of the square of the distance, it is good practice, however, if there are safety conditions for the patient, to ensure that the accompanying persons and the operators are positioned as far as possible from the primary beam. The radiologic technologists performing the examination shall wear suitable Individual Protection Device being usually exposed, during the calendar year, to other sources of ionizing radiations [18, 19].

## The “dose” communication and the appropriate patient information

An adequate communication of the benefits deriving from a radiological procedure, of the ionizing radiations (IR) dose and of the potential adverse effects, represents a key priority of the therapeutic relationship Clinician-Patient, and allows to avoid unjustified concerns and the necessary examinations refuse [30].

The parents often interact with the paediatricians or with the neonatologists asking explanations about the radiological procedures performed on the hospitalized new-borns in NICU. Nevertheless, it is not infrequent that they put the questions to the technicians or to the nurses. It is therefore important that all the operators have a certain familiarity in radioprotection, knowing the mean of the data they transmit to the patients and what represent, avoiding an “alarmistic” communication [30].

### Radiation protection basic notions

The ionizing radiations (IR) have been classified in class 1 by the WHO, inside the group of the substances surely carcinogenic for the humans. Nevertheless, the carcinogenic effect of the RI is shown in vivo only for doses > 100 mSv. Under this threshold (low doses) currently available scientific data do not allow to esteem the harmful effects of the IR for certain, that are stochastics (or probabilistic, not dose-related) and not distinguishable from those induced by other factors. In the low doses range, as reported in the publication 103 of the International Council of Radiation Protection (ICRP), the theory of the “linear effect without threshold” (LNT, linear-no-threshold) is underlined. Following this theory there is a direct linear relationship among the entity of the dose and the cell damage probability (not gravity) [31].

For these reasons a precautionary attitude is pursued in the justification principle respect according to which every single medical-radiological procedure must be preventively justified so that to guarantee that the benefits are superior to the risks [2, 3, 18, 19, 31].

When the benefits and the risks of a procedure are evaluated, it is not often considered the risk, not negligible, of a missed diagnosis or of a delay in the beginning of the cares with effects on the child outcome.

### How to establish an effective communication

In preparing an effective physician-patient communication, it is essential to identify some key messages to transmit, personalizing every single situation (potential diagnosis, the new-born’s prognosis, cumulative exposure) and preparing in advance the answers to the most frequent questions, suiting the language for the interlocutors and avoiding, if possible, scientific terms or statistics and complex numbers. In the majority of the cases parents are not interested to a numerical information about the dose but to the risk that can derive from it. Therefore it is useful to remember that the radiological procedures use the radiations ionizing as the mean to get diagnostic information on our organism and to protect our health, and that the human body is provided with physical and biological mechanisms designated to protect us from RI late effects [30].

In particular:

- Identify the exposure and the dose
- Compare the dose of the proposed procedure to other imaging techniques and the natural background
- Strongly underline the benefits and the clinical necessity of the procedure
- Ensure that the dose is personalized on the child dimension
- Avoid inducing worries explaining the potential risks from IR
- Use tables in which quantitative (i.e.0.01%) and qualitative (negligible for RX, very low for CT) estimations are reported and compare to basal tumour incidence (40–42%) and the other risks deriving from daily activities such as to cross the road or to drive a car

In the physician-patient communication it is necessary to pay attention in forwarding the information both in oral and figurative language. The same numerical data, if not correctly explained, may result unjustified alarming as in the use of “equivalent thorax examinations” (i.e. 1 TC = 200 RX; would you have 200 rx in the same time’) or quantitative estimations of neoplastic risk (i.e. 0.01% after RX), that are usually “personalized” by the parents especially in emotional stress situations (that 1/10000 will surely be my child!) [30, 31].

The figurative (and oral) language to avoid, a typical example is the fast exiting of the operators from the NICU whenever a radiography is performed, with sentences like “pay attention they are taking the rx” or “quickly get out”, creating unnecessary anxieties in the parents of the hospitalized children [31] (Tables 2 and 3).

**Table 2:** Practical example of alarmistic and ineffective communication

Question	Answer
Are there some risks?	Theoretical of neoplasia. There are no absolute certainties.
How much the risk is?	Very small (in theory)
If he/she will develop a tumour after this exposition?	We will never know if it has come for this examination or for another reason

**Table 3:** Practical example of an effective communication

Question	Answer
Why this exam?	We need some information that only this exam can give us in an accurate and rapid way.
Are there any risks?	The risk, if it exists, is very small and it is a possible radio-induced tumour. But we are not sure that really a risk exists to the RX and TC low doses.
When they may occur?	The most important risk is not to make an important diagnosis in the next minutes / times / days. The potential radio-induced damage may take some years
If he/she will develop a tumour after this exposition?	In Italy the basic level to develop a tumour is 40-42%. The additional risk is negligible (from 40-42% to 40.01-42.01%)
How can I know that the IR dose is the appropriate one for my child?	We use different protocols and techniques according to the clinical questions and of the child dimension. Then we periodically compare oud delivered doses with national and international references to be confident to work in a safety range.
This exam is really necessary?	Yes. I understand your worries for the potential effects of the RI but the examination is important for your child. If you think about it, every time you drive your car you don’t worry about the probabilities you have to be involved in a deadly accident, because the advantage to use the car is superior. This is the same thing.

## Conclusions

This document will allow a standardization of the approach to the exposures in NICU, although oriented to a flexible methodology.

## Data Availability

Not applicable.

## Appendix

### Execution and quality requirements

- **Percutaneous Central (CVC) Vein Catheterization**

*Verify the catheter’s position* (the top of the catheter must be between T3 and T5 if in superior part of the vena cava and between T8 and T10 if in inferior).

In case of catheter of small dimensions (1 Fr) it could be useful the use of a low osmolality contrast media that must be injected very slow in a quantity just enough to fill only the catheter.

The radiological exam must be repeated after 3–5 s to allow the blood flow to eliminate the excess of contrast media from the top of the catheter, in order to identify it [32].

- **Umbilical Vein Catheterization (UVC)**

Verify the UVC position (the top of the catheter must be far from the hepatic veins origin, from the portal vein and from the oval foramen; UVC must be inside the venous ductus or in VCI, 0.5–1 cm over the diaphragm, T8-T9).

The primary indications for the positioning of the catheter inside the umbilical vein, are the pharmacological reanimation in the delivery room, the need for a central access during the first days in critical patients for parenteral nutrition and drugs administration, or the necessity to perform an exchange-transfusion (EXT). Secondary indications are the infusion of solutions, drugs and the need of blood tests. Possible side effects to the positioning of an umbilical (UVC) venous catheter are omphalitis, omphalocele, necrotizing enterocolitis (NEC), peritonitis or damage from the infusion extravasation [33].

- **Umbilical Artery Catheterization (UAC)**

Low position (L3-L5, under inferior mesenteric artery), high position (T6-T9, thoracic aorta, over the celiac-mesenteric-renal arteries) [34].

- **Endotracheal Intubation**

Calculate the distance from tracheal carina (position: middle bystander of the trachea, 1–2 cm over the carina, T4 level) and be sure that the right principal bronchus has not been intubated and that there are no lobar hypoventilation phenomena [34].

- **Nasogastric probe**

Make sure that the top is projected inside the stomach [34].

- **Thoracic drain**

The apposition of the catheter for the thoracic drain could be necessary in case of pneumothorax or pleural effusion in order to guarantee a proper pulmonary expansion. Position: mid-axillary, 4°-6° intercostal space. Orientation: apical anterior or antideclivous in case of pneumothorax, basal declivous in case of effusion [35].

## Abbreviations

AIFM: Italian Association of Physics in Medicine
Al: aluminum
AP: anterior-posterior projection
BPD: bronchopulmonary dysplasia
CDH: congenital diaphragmatic hernia
CPAM: congenital pulmonary airway malformation
CR: computed-radiography
Cu: cupper
DAP: dose-area product
DFR: focus-receptor distance
DR: digital-radiography
HPS: hypertrophic pyloric stenosis
ICRP: International Council of Radiation Protection
IR: ionizing radiations
LDR: diagnostic reference levels
LL: latero-lateral projection
LNT: linear-no-threshold
MAS: Meconium aspiration syndrome
NEC: necrotizing enterocolitis
NICU: Neonatal Intensive Care Units
NRDS: Neonatal respiratory distress syndrome
PIE: pulmonary interstitial emphysema
SIN: Italian Neonatology Society
SIP: Italian Paediatric Society
SIRM: Italian Medical Radiology Society
TEF: tracheoesophageal fistula
TTN: Transient tachypnoea of the new-born
